# Association between nutritional status and subjective health status in chronically ill children attending special schools

**DOI:** 10.1007/s11136-015-1130-4

**Published:** 2015-09-11

**Authors:** Koen Joosten, Kelly van der Velde, Pieter Joosten, Hans Rutten, Jessie Hulst, Karolijn Dulfer

**Affiliations:** Department of Pediatrics, Pediatric Intensive Care, Erasmus MC - Sophia Children’s Hospital, Dr. Molewaterplein 60, 3015 GJ Rotterdam, The Netherlands; Department of Pediatrics, Erasmus MC - Sophia Children’s Hospital, Rotterdam, The Netherlands; VUmc School of Medical Sciences, Amsterdam, The Netherlands; Ministry of Economic Affairs, The Hague, The Netherlands; Department of Pediatric Gastroenterology, Erasmus MC - Sophia Children’s Hospital, Rotterdam, The Netherlands; Department of Child and Adolescent Psychiatry/Psychology, Erasmus MC - Sophia Children’s Hospital, Rotterdam, The Netherlands

**Keywords:** Prevalence malnutrition, Chronic disease, Subjective health status, Paediatrics, Quality of life, Nutritional status

## Abstract

**Purpose:**

In hospitalized children with a chronic disease, malnutrition was associated with a lower subjective health status. In outpatient children with a chronic disease attending special schools, this association has never been studied. The aim of this study was to assess the association between nutritional status and subjective health status in chronically ill children attending special schools.

**Methods:**

Overall, 642 children, median age 9.8 years (IQR 7.7–11.5), 60 % male, 72 % Caucasian, were included in this prospective study in nine special schools for chronically ill children in the Netherlands. Overall malnutrition was assessed as: acute malnutrition (<−2 SDS for weight for height (WFH)) and chronic malnutrition (<−2 SDS for height for age). The malnutrition risk was assessed with the nutritional risk-screening tool STRONG_kids_. Subjective health status was assessed with EQ-5D.

**Results:**

Overall, 16 % of the children had overall malnutrition: 3 % acute and 13 % chronic malnutrition. Nurses reported ‘some/severe problems’ on the health status dimensions mobility (15 %), self-care (17 %), usual activities (19 %), pain/discomfort (22 %), and anxiety/depression (22 %) in chronically ill children. Their mean visual analogue scale score (VAS) was 73.0 (SD 11.1). Malnutrition, medication usage, and younger age explained 38 % of the variance of the VAS score.

**Conclusions:**

The presence of overall malnutrition in chronically ill children attending special schools was associated with lower subjective health status, especially in younger children and in those with chronic medication usage. Therefore, it is important to develop and use profile-screening tools to identify these children.

**Electronic supplementary material:**

The online version of this article (doi:10.1007/s11136-015-1130-4) contains supplementary material, which is available to authorized users.

## Introduction

In the Netherlands, approximately 300,000 children have a chronic disease: this is 10 % of all children [1]. When admitted to a hospital, these children with a chronic disease have a higher risk of malnutrition than previously healthy children [2]. Moreover, these children have a higher prevalence of chronic malnutrition compared with those without an underlying disease [3]. Malnutrition may adversely affect physical, mental, and social aspects of the child’s health [4–6]. Possible effects of malnutrition on physical health are impaired growth, lowered immunity, and even mortality [7, 8]. Malnutrition has also been associated with impaired development of executive functions and lower school achievements [5, 6]. In addition, it may also be related to an increased incidence of attention deficit problems, aggressive behaviour [9], and an increased risk of emotional, social, and cognitive impairments [10]. Moreover, children and adolescents with a chronic condition may report an impaired subjective health status compared with healthy peers [11].

After hospitalization, chronically ill children may suffer from sequelae, even though their chronic disease is stable. In the Netherlands, most children with a chronic disease will attend regular primary schools at the age of 4 years. However, those children with sequelae, necessitating medical care, may need to attend special schools for chronically ill children. Jelsma and Ramma [12] reported that children with functional impairments in special schools, ranging from learning disabilities to movement disorders, reported more problems on the mobility and self-care dimensions, compared with children at regular schools.

Because of the medical sequelae in chronically ill children in special schools, there is a higher risk of malnutrition, and therefore, it is important to increase awareness about the nutritional status and practices in these children. Since it was recently shown that malnutrition was associated with a lower subjective health status in hospitalized chronically ill children [13], it might be hypothesized that malnutrition in chronically ill children attending special schools is also associated with a lower subjective health status.

The first aim of our study was to screen the nutritional status and subjective health status of chronically ill children attending special schools. Secondly, we investigated the association between nutritional status, risk of malnutrition, and subjective health status in these children.

## Methods

### Assessment procedure

Nine primary schools for chronically ill children throughout the Netherlands were invited to voluntarily participate in this study. The screening of the children took place between December 2010 and January 2011. There were no exclusion criteria. Because of the standard nature of the measurements taken regularly by the nurses of these schools, waived informed consent was obtained from our local medical ethical commission (MEC-2015-060). Parents or caregivers were asked for permission through a letter and could refrain from participation without consequences. Two nurses at each school assessed child characteristics, nutritional status, risk of developing malnutrition, and subjective health status in the children.

**Fig. 1:**
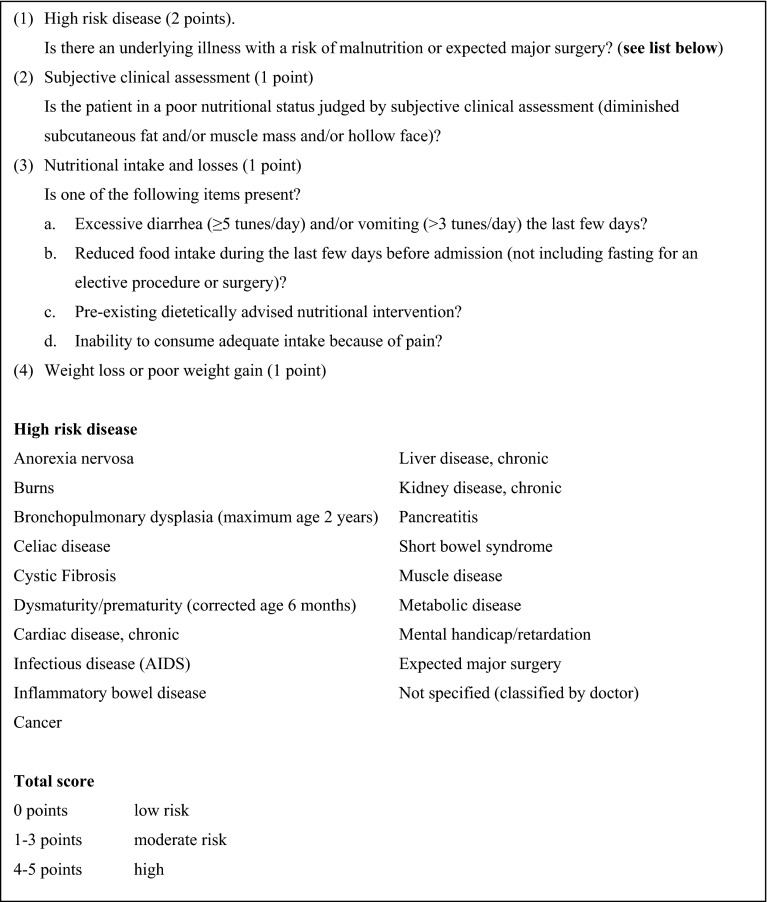
Nutritional risk-screening tool STRONG_kids_

For all children, age, gender, and diagnosis concerning chronic illness were recorded. The chronic illness was classified as neurologic, respiratory, endocrine, cardiac, metabolic, gastrointestinal, renal, oncological, multiple diagnostic groups, and others. For analysis, these types of chronic illness were classified according to the first question of the nutritional risk-screening tool STRONG_kids_ (see Fig. 1). Furthermore, the onset of the child’s chronic disease was reported as: the chronic disease was present at birth or the child developed the chronic disease during life. Children were classified with or without a syndromal disorder. Ethnic background was classified as Caucasian and non-Caucasian.

### Instruments

#### Nutritional status

In order to assess nutritional status, anthropometric measurements of standing weight and height were obtained. Beforehand, all participating schools were instructed to collect all data with a standardized assessment, using standard equipment (digital scales, stadiometer) [14]. Anthropometric data from Dutch children were compared with published standards based on a Dutch reference population [15]. Data of children with a Turkish or Moroccan background were compared with available culture-specific growth charts [15]. For syndromal children, specific growth references were used if available [16]. All data were translated into standard deviation scores (SDS), resulting in weight for age (WFA), weight for height (WFH), and height for age (HFA).

#### Malnutrition

Acute malnutrition was indicated as a WFH SDS score <−2. Chronic malnutrition was indicated as a HFA SDS score <−2. Overall malnutrition was defined as the presence of acute malnutrition and/or chronic malnutrition [17].

The risk of malnutrition was assessed with the nutritional risk-screening tool STRONG_kids_ (Screening Tool for Risk on Nutritional status and Growth) developed for hospitalized children [18]. This nutritional risk-screening questionnaire consists of four items, and each item is allocated a score of 1 or 2 points with a maximum total score of 5 points (Fig. 1). Questions answered ‘unclear’ were classified as ‘no’. A child with a STRONG_kids_ risk score of 0, 1–3, or 4–5 has, respectively, a low, moderate, or high risk of developing malnutrition. There are no data available on the sensitivity or specificity of the STRONG_kids_ tool.

#### Subjective health status

The generic EQ-5D was used to assess subjective health status [19]. It comprises a descriptive system with five dimensions: mobility, self-care, usual activities, pain/discomfort, and anxiety/depression. Each dimension has three levels: no problems, some problems, and severe problems. This descriptive system of the EQ-5D was combined with the visual analogue scale (VAS). The VAS represents the child’s health in the past week, resulting in a score between 0 and 100, with a higher score representing a better health status.

### Statistical analysis

Descriptive analyses were used to describe the study population. WFA, HFA, and WFH deviations from 0 were carried out using one-sample *t* tests and were represented as standard deviation scores (SDS). Since age was not normally distributed, comparison of continuous age scores between children with malnutrition versus those without malnutrition was completed with Mann–Whitney *U* test. Pearson’s Chi-square tests were used to test differences in nominal and ordinal distributions between children with malnutrition versus those without malnutrition of: gender, ethnic background, disease present at birth, and prediction of malnutrition (low risk versus moderate/high risk).

EQ-5D scores were presented as a health profile, using frequencies of reported problems for each level of each dimension. For each dimension, the number of ‘severe problems’ was very low. Therefore, the EQ-5D levels were dichotomized into ‘no problems’ (level 1) versus ‘some/severe problems’ (level 2 and 3 together). Chi-square tests were used to compare EQ-5D percentages between groups for: malnutrition, risk of malnutrition, chronic disease present at birth, and medication usage. Between these groups, differences in continuous visual analogue scales (VAS) were tested with unpaired *t* tests. Besides, Pearson’s correlation between VAS score and age was assessed.

To determine the association between malnutrition and subjective health status (continuous VAS scores), first univariate regression analysis was applied. Since VAS scores differed significantly between special schools, these univariate analyses were corrected for site of data collection (transformed as dummy variables). After this, all variables that were significantly associated with VAS scores were forced simultaneously into a multiple regression analysis. Variables that were not significant (*p* > .050) in the final model were removed (backward elimination procedure), and then, the total explained variance (*R*^2^) was calculated. Site of data collection was forced into the models.

As to the assumptions of multiple regression analysis, we first checked for multicollinearity and calculated the variance inflation factor (VIF). For each model, the average of the VIF’s of the entered functional health variables was around one, which is expedient. The linearity assumption was examined with a plot of the standardized residuals on the *Y*-axis and the standardized predicted values on the *X*-axis. No pattern was discovered in this scatterplot. To check normality, the histogram of the regression-standardized residuals was inspected. This histogram was normally distributed. Statistics were conducted using SPSS version 21.0, and a two-sided *p* value of <0.05 was considered significant.

## Results

### Baseline characteristics

**Fig. 2:**
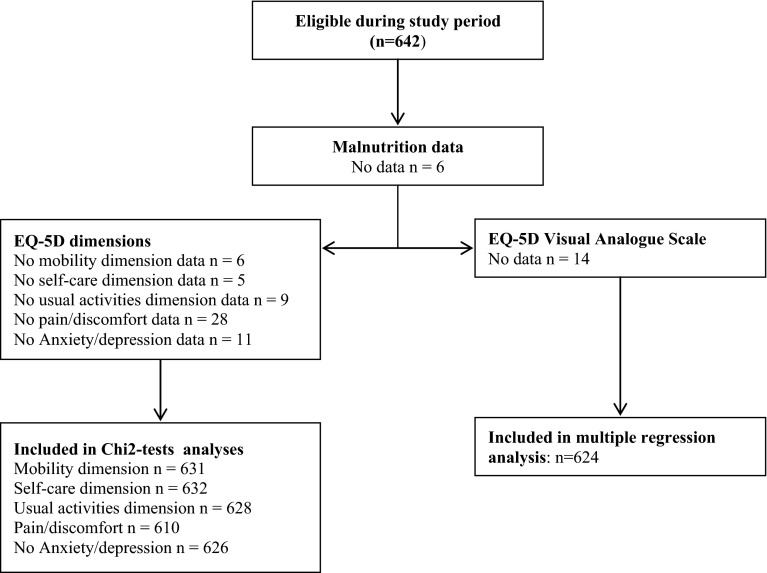
Study enrolment

A total of 642 children were included from nine schools, with a median of 70 children participating from each school (range 39–109), see Supplemental Table A for number of children in each school. Because of the standard nature of the measurements that are taken regularly by the nurses of these schools, all children who were present between December 2010 and January 2011 were included (see Fig. 2). The median age was 9.8 years (IQR 7.7–11.5), 60 % were male, and 71.8 % were Caucasian (see Table 1). Medication use was reported in 419 children (65.3 %) including 106 children using corticosteroids, 25 children using antibiotics, and 15 children using diuretics. Weight and height were available in 99.2 and 99.4 % children, respectively. The mean SDS for WFA (−0.16), WFH (0.41), and HFA (−0.63) all significantly differed from 0 (WFH and HFA *p* ≤ 0.001; WFA *p* ≤ 0.01).

**Table 1:** Baseline characteristics, malnutrition, and subjective health status

	*n* = 642
Child characteristics	
Gender, boy, n (%)	385 (60)
Median age (range)	9.8 (4.2–13.4)
Ethnic background:	
White, *n* (%)	461 (72)
Disease characteristics, *n* (%)	
High-risk disease, yes, *n* (%)^a^	193 (30)
Medication usage, yes, *n* (%)	419 (65)
Steroids	106 (17)
Antibiotics	25 (4)
Diuretics	15 (2)
Other	273 (42)
Disease present at birth, *n* (%)	
Yes	250 (38.9)
No	218 (34.0)
Missing	174 (27.1)
Sick leave last 3 months (*n* = 573), yes	334 (58)
Median days sick last 3 months (IQR)	1 (0–4)
*Nutritional status*	
Malnutrition, *n* (%)	
Chronic malnutrition	88 (13.8)
Acute malnutrition	19 (3.0)
Overall malnutrition	102 (16.0)
Prediction of malnutrition, *n* (%)	
Low risk	381 (59.3)
Moderate risk	247 (38.5)
High risk	14 (2.2)
*Subjective health status*	
Some/severe problems with, *n* (%)	
Mobility	96 (15.1)
Self-care	106 (16.6)
Daily activities	118 (18.6)
Pain/discomfort	137 (22.3)
Anxiety/depression	139 (22.0)
Visual analogue scale, *m* (sd)	73.0 (11.1)

^a^High-risk disease based on first question of nutritional risk-screening tool STRONG_kids_ (see Fig. 1)

### Malnutrition

The prevalence of children with acute malnutrition was 3.0 %. The prevalence of chronic malnutrition was 13.8 %. Since almost 1 % of the children were both acute and chronically malnourished, the overall prevalence of malnutrition was 16.0 % (see Table 1). No differences in malnutrition were found between boys and girls or between Caucasian and non-Caucasian children.

The year of onset of disease was known in 468 children. The median duration of illness in these children was 7 years (IQR 5—10 years). In 250 children (53.4 %), the disease was present at birth. The prevalence of acute, chronic, and overall malnutrition in these 250 children was 4.0, 18.8, and 21.2 %, respectively. These were significantly higher than the prevalence among children who became chronically ill later in life (0.9, 8.8, 9.7 %; all *p* < 0.05). The prevalence of malnutrition in the various diagnostic groups is reported in supplemental Table B. The highest prevalence of chronic and overall malnutrition was found among children with a metabolic disease, while the prevalence of acute malnutrition did not differ.

### Risk of malnutrition (STRONG_kids_)

The four items of the STRONG_kids_ questionnaire, i.e. ‘high-risk disease’, ‘subjective clinical assessment’, ‘nutritional intake and losses’, and ‘weight loss or poor weight gain’, were scored with ‘yes’ in 30.1, 12.3, 10.1, and 2.6 % of the children, respectively. Based on the STRONG_kids_ questionnaire, 59.3 % of the children were classified in the low-risk group, 38.5 % in the moderate-risk group, and 2.2 % in the high-risk group for developing malnutrition. The highest prevalence of acute, chronic, and overall malnutrition was found in the high-risk group, though there was no significant difference between the moderate- and high-risk group (see supplemental Table C).

### Subjective health status

Overall, chronically ill children reported ‘some problems’ or ‘severe problems’ on the health status dimensions mobility (15 %), self-care (17 %), usual activities (19 %), pain/discomfort (22 %), and anxiety/depression (22 %). Their mean VAS score was 73.0 (SD 11.1). Higher age was significantly positively associated with a higher VAS score, *r* = .21, *p* < .01.

In Table 2, the counts and percentages of subjective health status problems in children with and without malnutrition, with risk of malnutrition versus those without risk, with their chronic disease present at birth versus those whose disease developed later in time, and for those children with medication usage versus those without medication usage are presented. For children with overall malnutrition, nurses reported significantly more often some/severe problems on the dimensions mobility (24 %) and self-care (24 %) than for children without overall malnutrition (13 and 15 %, respectively) (see Table 2). Nurses also reported more often some/severe problems on the dimensions mobility (19 %), self-care (23 %), and usual activities (23 %) for children whose chronic disease was present at birth, compared with those whose chronic disease developed later in life (respectively, 10, 10, and 19 %).

**Table 2:** Chi-square comparisons between subjective health status problems for malnutrition, prediction of malnutrition, chronic disease present at birth, and medication usage

Problems	Subjective health status problems									
	Mobility *n* (%)		Self-care *n* (%)		Usual activities *n* (%)		Pain/discomfort *n* (%)		Anxiety/depression *n* (%)	
	No	Some/severe	No	Some/severe	No	Some/severe	No	Some/severe	No	Some/severe
Malnutrition										
No	458 (87)^a^	71 (13)^a^	450 (85)^b^	81 (15)^b^	428 (81)	98 (19)	399 (78)	113 (22)	411 (78)	113 (22)
Yes	78 (76)^a^	24 (24)^a^	77 (76)^b^	24 (24)^b^	83 (81)	19 (19)	75 (77)	23 (23)	77 (75)	25 (25)
STRONG_kids_										
No risk	327 (87)	50 (13)	322 (85)	55 (15)	317 (84)^c^	59 (16)^c^	300 (83)^d^	63 (17)^d^	297 (80)	76 (20)
Moderate/high	231 (83)	46 (17)	209 (80)	51 (20)	198 (77)^c^	59 (23)^c^	177 (71)^d^	74 (29)^d^	195 (76)	63 (24)
Chronic disease present at birth										
No	194 (90)^e^	21 (10)^e^	195 (90)^f^	21 (10)^f^	193 (81)^g^	22 (19)^g^	170 (81)	40 (19)	167 (78)	47 (22)
Yes	202 (81)^e^	46 (19)^e^	190 (77)^f^	58 (23)^f^	188 (77)^g^	56 (23)^g^	178 (76)	56 (24)	202 (82)	43 (18)
Medication usage										
No	183 (83)	37 (17)	179 (81)	43 (19)	176 (80)	44 (20)	181 (84)^h^	34 (16)^h^	184 (84)^i^	35 (16)^i^
Yes	357 (86)	59 (14)	352 (85)	63 (15)	339 (82)	74 (18)	296 (74)^h^	103 (26)^h^	308 (75)^i^	104 (25)^i^

STRONG_kids_: a nutritional screening tool to assess the risk of malnutrition

^b,c,d^The observed counts differed significantly from the expected counts under the assumption of no association: * *p* < 0.05

^a,e,f,g,h,i^The observed counts differed significantly from the expected counts under the assumption of no association: * *p* < 0.01

For children who were at risk of developing malnutrition (STRONG_kids_), nurses reported more some/severe problems on usual activities (23 %) and pain/discomfort (29 %), compared with those children who were not at risk (respectively, 16 and 17 %). Nurses also reported more pain/discomfort problems (26 %) in children who used medication compared with children who did not use medication (16 %). Nurses also reported more anxiety/depression problems in 25 % of the children who used medication versus in 16 % of the children who did not use medication.

Children with overall malnutrition obtained a lower VAS score than children without overall malnutrition *M* = 70.6 (SD = 11.3) versus *M* = 73.6 (SD = 11.0), *t*(624) = 2.46, *p* < .02. As to the *risk* of malnutrition, those children with a low risk obtained a higher VAS score compared with those with a moderate/high risk *M* = 74.2 (SD = 11.0) versus *M* = 71.4 (SD = 11.1), *t*(626) = 3.11, *p* < .01. For children whose chronic illness was present at birth, a significantly lower VAS score was reported compared with those who developed their chronic illness during life *M* = 72.3 (SD = 9.9) versus *M* = 75.6 (SD = 10.9), *t*(453) = 3.31, *p* < .01. Nurses reported a significantly lower mean VAS score in children who used medication than in children who did not *M* = 71.8 (SD = 10.7) versus *M* = 75.5 (SD = 11.5), *t*(626) = 4.07 *p* < .01.

### Associations between nutritional status and subjective health status

In univariate regression analysis, corrected for the different special schools, malnutrition in chronically ill children was significantly associated with worse subjective health status; explaining 35 % of its variance (see Table 3). In multivariate regression analysis, all variables that significantly differed between children with malnutrition and those without malnutrition (disease present at birth, risk of developing malnutrition (STRONG_kids_), medication usage, and age in years) were forced simultaneously into the model. Corrected for the different special schools, dichotomized risk of developing malnutrition (STRONG_kids_) and disease present at birth were no longer significantly associated with VAS scores (*p* = .33). Medication usage and younger age, together with malnutrition, explained 38 % of the variance of worse subjective health status.

**Table 3:** Univariate and multivariate models of significant associations between subjective health status (visual analogue scale) and malnutrition

	Constant	Unstandardized *β*	SE	Standardized *β*	*p* value	Multiple *R* ^2^
Model 1^a^						
Malnutrition (1 = yes)	69.5	−2.9	1.2	−.10	<.01	0.35
Model 2^a^						
Malnutrition (1 = yes)	63.0	−2.9	1.0	−.10	<.01	0.38
Medication (1 = yes)		−2.1	0.8	−.09	<.01	
Age in years		0.8	0.2	.17	<.01	

Model 1: *F*(9,614) = 36.1, *p* < .001, *R* ^2^ = .35, model 2: *F*(11,612) = 33.7, *p* < .001, ∆*R* ^2^ = .03, *p* < .001

^a^Corrected for site of special school

## Discussion

The purpose of this study was to determine the associations between malnutrition, the risk of developing malnutrition, and subjective health status in children attending special schools for chronically ill children. The prevalence of acute, chronic, and overall malnutrition in our sample was 3.0, 13.8, and 16.0 %, respectively. Compared with the reported prevalence of acute and chronic malnutrition (11 and 9 %) in the Dutch hospitals, the prevalence of acute malnutrition was lower and chronic malnutrition was higher. This may be associated with the absence of acute illness in our study population in special schools, compared with hospitalized children [3, 18].

### Subjective health status dimensions

Overall malnutrition was associated with more nurses-reported problems on mobility (capacity for walking about) and self-care (capacity for self-care in terms of washing and dressing). In addition, a moderate or high risk of *developing* malnutrition (assessed with the screening tool STRONG_kids_) was associated with more problems on usual activities (attending school, sport and leisure activities, play, do things with family or friends) and pain/discomfort (to what extent the child experienced pain or discomfort). Disease characteristics such as the presence of the chronic disease at birth and medication usage were also associated with several subjective health dimensions. Remarkably, only chronic medication use was associated with more problems on anxiety/depression dimension (to what extent the child experienced anxious or depressed feelings).

In chronically ill children who are attending special schools, regular measurements of weight and height is standard care. Using these measurements to calculate the SD scores for weight and height and thereby identifying those children with acute (WFH SD scores <−2) and chronic (HFA SD scores <−2) malnutrition might also help to identify children who are at risk of developing problems on subjective health status dimensions. Besides, determination of the risk of malnutrition using a nutritional screening tool like STRONGkids might also be helpful in identifying at-risk children. This is the first study in which the STRONG_kids_ was applied in a non-clinical setting, and it seemed to be a practical, easy, and reliable tool for assessment of nutritional risk and subjective health status.

### Subjective health status visual analogue scale

Children with a chronic disease in our sample obtained a lower VAS score (*M* = 73.0) for subjective health compared with children from previous studies with autism spectrum disorder (*M* = 80.7), with intellectual disorders (*M* = 79.4), with movement disorders (*M* = 76.9), and with hearing disorders (*M* = 88.1) [20]. They also obtained lower VAS scores compared with children with disabilities, ranging from learning disabilities to movement disorders, attending special schools (*M* = 88.4) [12]. In these two studies, children (who were actually older) reported their own subjective health status, whereas in our study, nurses employed at the special schools assessed the subjective health status of the children. Since in our study children were younger and higher age was associated with a higher VAS score, this may also explain the differences in VAS scores between the studies.

In our study, overall malnutrition, medication usage, and age explained almost 40 % of the variance of the subjective health status VAS. In practice, these variables are easily assessable and may identify those children with a chronic disease, at admission to the special school, who are at risk of a low subjective health status. Overall malnutrition was the sole ‘predictor’ variable in this study that might be influenced with an intervention. Therefore, it might be useful to develop school-feeding intervention programmes. These programmes may help to ameliorate and prevent some of the subjective health status problems. A recent Cochrane review concluded that school meals might have a number of small benefits for children attending regular schools. In younger children, school meals resulted in small improvements in weight, height, school attendance, math performance, and behaviour. Besides, it may have an impact on intelligence scores [21]. For future research, it might also be useful to assess the influence of school-feeding programmes on subjective health status in chronically ill children attending special schools.

### Strengths and limitations

This is the first study in a large sample of chronically ill children attending special schools, in which associations between malnutrition, risk of malnutrition, and subjective health were assessed. Moreover, nine out of 11 special schools for chronically ill children in the Netherlands participated in this study, so it is a nationwide study.

Although very few children in these schools did not participate, we had no background information on these non-participating children, so bias may have played a small role. Another limitation is the fact that the one-dimensional EQ-5D questionnaire was used to assess proxy-reported subjective health status instead of a self-reported multidimensional subjective health status measure. For future research, perhaps assessing both self-reported and proxy-reported quality of life, including an emotional reaction to problems, could be more informative.

## Conclusion

Malnutrition in chronically ill children attending special schools was associated with lower subjective health status. Especially children who have their illness from birth, who used medication and the youngest children were at risk of a lower subjective health status. Therefore, it is important to identify these children using profile-screening tools. School-feeding programmes probably can improve the nutritional status of these children which may also have a positive effect on the subjective health status in these children.

## Electronic supplementary material

Below is the link to the electronic supplementary material.

Supplementary material 1 (DOCX 14 kb)
