# Supplementary material for: Association between nutritional status and subjective health status in chronically ill children attending special schools
Source: Qual Life Res. 2015 Sep 11;25:969–77. doi: 10.1007/s11136-015-1130-4 (PMC4830861; doi:10.1007/s11136-015-1130-4)
Supplement: Supplementary file 1 — Supplementary material 1 (DOCX 14 kb) [file 11136_2015_1130_MOESM1_ESM.docx]

**Supplemental Table A: Prevalence and mean VAS scores of assessed children classified by Special School**

| Name of the school | Frequency | Percent | Mean VAS* |
| --- | --- | --- | --- |
| A. Schweitzerschool | 76 | 11,8 | 69.1 (8.4) |
| Coronelschool | 86 | 13,4 | 73.0 (6.2) |
| W.B. Noteboomschool | 68 | 10,6 | 69.5 (6.2) |
| Reconvalescentenschool | 39 | 6,1 | 66.8 (12.6) |
| Schutte's Bosschool | 108 | 16,8 | 69.2 (7.3) |
| Groninger Buitenschool | 47 | 7,3 | 61.2 (8.6) |
| Openlucht school Rotterdam | 39 | 6,1 | 79.6 (1.8) |
| Openlucht school Breda | 109 | 17,0 | 81.9 (11.1) |
| De Schaus | 70 | 10,9 | 81.0 (13.5) |
| Total | 642 | 100,0 | 73.1 (11.1) |

* VAS = Visual Analogue Scale, schools differed significantly F(8,619)=39.0, p<0.001

**Supplemental Table B: Malnutrition classified by diagnosis**

| Diagnosis | Acute  malnutrition (%) | Chronic  malnutrition (%) | Overall  malnutrition (%) |
| --- | --- | --- | --- |
| Neurologic (n=150)  Respiratory (n=101)  Endocrine (n=60)  Cardiac (n=32)  Metabolic (n=28)  Gastro-intestinal (n=27)  Renal (n=20)  Oncologic (n=12)  Multiple-diagnostic groups (n=77)  Others (n=73)  None (n=17)  Syndromal (n=45) | 4,0  1,0  0,0  3,1  7,1 ^a^  3,7  0,0  0,0  5,2  4,1  5,9  0,0 | 15,3  11,9  11,7  18,8  28,6 ^b^  14,8  20,0  16,7  11,7  6,8  11,8  13,3 | 18,0  12,9  11,7  21,9  35,7 ^c^  14,8  20,0  16,7  15,6  9,6  17,6  13,3 |

Acute malnutrition: weight-for-height < -2 SD; chronic malnutrition height-for-age < -2 SD; overall malnutrition: acute and/or chronic malnutrition.

*^a^ = highest % acute malnutrition, not significant*

*^b^ = highest % chronic malnutrition, sign. higher than respiratory and others (p<0,05)*

*^c^ = highest % overall malnutrition, sign. higher than respiratory, neurologic, syndromal, endocrine, others and multiple (p<0,05).*

**Supplemental Table C: Prevalence of malnutrition classified by STRONG_kids_ risk group**

|  | Low risk | Moderate risk | High risk |
| --- | --- | --- | --- |
| Acute malnutrition (%) ^a, b^  Chronic malnutrition (%) ^a^  Overall malnutrition (%) ^a, b^ | 1,1  10,8  11,1 | 5,3  17,6  22,1 | 14,3  28,6  42,9 |

*^a^ Significant difference between the low and moderate risk group.*

*^b^ Significant difference between the low and high risk group.*
